# Sirtuins Affect Cancer Stem Cells via Epigenetic Regulation of Autophagy

**DOI:** 10.3390/biomedicines12020386

**Published:** 2024-02-07

**Authors:** Ferenc Sipos, Györgyi Műzes

**Affiliations:** Immunology Division, Department of Internal Medicine and Hematology, Semmelweis University, 1088 Budapest, Hungary; muzes.gyorgyi@med.semmelweis-univ.hu

**Keywords:** sirtuins, SIRT, acetylation, deacetylation, cancer stem cells, autophagy, epigenetics, DNA methylation

## Abstract

Sirtuins (SIRTs) are stress-responsive proteins that regulate several post-translational modifications, partly by acetylation, deacetylation, and affecting DNA methylation. As a result, they significantly regulate several cellular processes. In essence, they prolong lifespan and control the occurrence of spontaneous tumor growth. Members of the SIRT family have the ability to govern embryonic, hematopoietic, and other adult stem cells in certain tissues and cell types in distinct ways. Likewise, they can have both pro-tumor and anti-tumor effects on cancer stem cells, contingent upon the specific tissue from which they originate. The impact of autophagy on cancer stem cells, which varies depending on the specific circumstances, is a very intricate phenomenon that has significant significance for clinical and therapeutic purposes. SIRTs exert an impact on the autophagy process, whereas autophagy reciprocally affects the activity of certain SIRTs. The mechanism behind this connection in cancer stem cells remains poorly understood. This review presents the latest findings that position SIRTs at the point where cancer cells and autophagy interact. Our objective is to highlight the various roles of distinct SIRTs in cancer stem cell-related functions through autophagy. This would demonstrate their significance in the genesis and recurrence of cancer and offer a more precise understanding of their treatment possibilities in relation to autophagy.

## 1. Introduction

SIRTs, which comprise the Silent Information Regulator 2 family of ancient proteins, are ubiquitous throughout all domains of life [1]. Seven SIRTs characterize mammals, numbered SIRT1 through SIRT7. SIRTs are distinctive post-translational modification enzymes that use NAD+ as a co-substrate to eliminate acyl groups from lysine residues. SIRTs display an effect on a wide range of substrates and significant metabolic processes. SIRTs target a broad spectrum of cellular proteins for post-translational modification via acetylation (e.g., SIRT1, 2, 3, and 5) or ADP-ribosylation (SIRT4 and 6). SIRT7 regulates the transcription of RNA polymerase I [2]. A diverse range of lipid acyl groups (e.g., acetyl, glutaryl, malonyl, succinyl, or long-chain acyl groups) are transferred from substrate proteins to the ADP-ribose portion of NAD+ via the distinctive NAD+-dependent protein deacylase activity of SIRTs [1,3,4,5,6]. Due to the critical significance of NAD+ in the cellular metabolism, SIRTs are able to regulate the function of a wide variety of protein substrates (including histones, transcription factors, metabolic enzymes, and cell membrane proteins) exclusively relying on NAD+.

**Figure 1:**
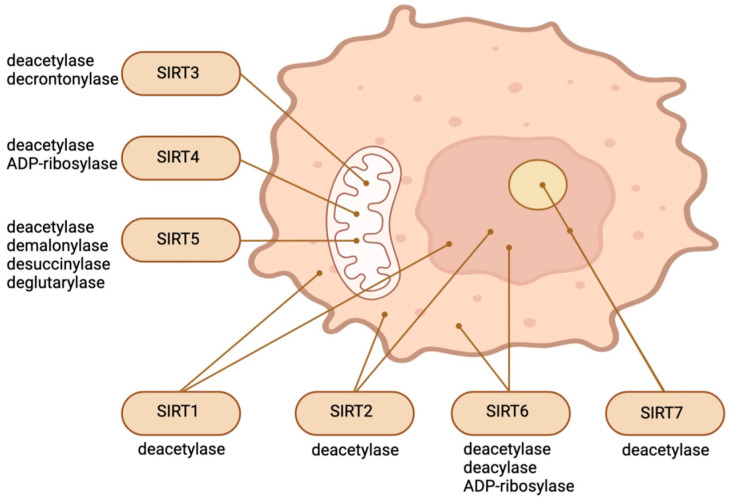
Intracellular location and main enzymatic activities of SIRTs. The figure was partly created with BioRender.com.

SIRT1, the most conserved SIRT in mammals, is predominantly localized in the nucleus but can transition between the cytosol and nucleoplasm in response to various environmental stimuli [7,8]. SIRT6 and SIRT7 are also nuclear proteins. SIRT7 is significantly enriched in the nucleolus, whereas SIRT6 is a chromatin-bound protein which is localized within the nucleus [9,10]. SIRT3, SIRT4, and SIRT5, three additional mammalian SIRTs, are located in the mitochondrial matrix, where they are involved in a variety of survival and metabolic processes associated with mitochondrial activity [11,12]. A considerable segment of SIRT5 is located outside of mitochondria, in the cytosol. It regulates glycolysis by demalonylating essential glycolytic enzymes. SIRT2, a protein which can also migrate between the cytosol and the nucleus, is predominantly cytosolic [11,12]. Existing research has focused extensively on the significance of SIRTs in relation to aging, age-related conditions and diseases, and nutrient sensing [13,14]. However, recent findings indicate that this family of distinctive enzymes also plays a crucial role in regulating stem cell biology [1]. Different SIRTs have different effects on SCs depending on the type of cell or stage in response to different environmental stimuli, even though they all depend on cellular NAD+. Embryogenesis and pluripotent SCs can be regulated by nuclear and cytosolic SIRTs through various mechanisms, including epigenetics, redox homeostasis, metabolism regulation, cellular stress response, and the control of pluripotency factors. SIRTs safeguard against stress-induced and aging-induced depletion of adult stem cells by preserving their capacity for self-renewal, quiescence, and regeneration [1]. Figure 1 illustrates the main enzymatic functions and intracellular location of SIRTs.

In light of the significance and far-reaching consequences associated with SIRTs’ activity, it is critical to comprehend the factors that govern their expression and activity. SIRT1, the most thoroughly investigated constituent of the SIRT family, has demonstrated regulation across various levels. While the precise mechanisms governing other SIRTs remain poorly comprehended, it seems that they operate within a regulatory environment that is similarly complex [15]. SIRT regulatory factors can be classified into four main categories [15,16,17]: (1) Regulating the transcription of multiple SIRTs can be achieved by modulating their nucleocytoplasmic transport. CCAAT-enhancer-binding protein-α and E2F transcription factor 1 promote the transcription of SIRT1, whereas p53 and Hypermethylated in cancer 1 inhibit it [15]. (2) The levels of available NAD substrate have an impact on the activity of SIRTs. AMP-activated protein kinase increases the expression of nicotinamide phosphoribosyl transferase to control NAD levels [15]. (3) SIRT activity can be changed at the protein level. SIRT1, for instance, is activated via a protein–protein interaction when the active regulator of SIRT1 binds directly to it, and sumoylation occurs at lysine 734. In contrast, direct binding with Deleted in Breast Cancer 1 (DBC1) inhibits SIRT1. Additionally, the post-translational modification mechanisms of SIRTs are diverse. The N- and C-terminal regions of the various SIRTs, which extend beyond the catalytic domains, mediate this regulation. These extensions are subject to various post-translational modifications, such as methylation, sumoylation, phosphorylation, and proteolytic cleavage [15,16]. (4) Additionally, the half-life of SIRT mRNAs is regulated. Binding the human antigen R RNA-binding protein to SIRT1 mRNA, for instance, prolongs the half-life of the latter. On the contrary, the implementation of miR-34a, miR-132, or miR-217 into SIRT1 leads to the translational repression of translation [15,16]. In addition, natural food products contain SIRT-activating compounds, which are small molecules responsible for regulating the expression of SIRTs. These are catechins, resveratrol, quercetin, berberine, fisetin, and curcumin [17].

## 2. SIRTs in the Epigenetic Regulation of Stem Cells

SIRT1. In comparison to adult tissues and cells, SIRT1 is significantly overexpressed in preimplantation embryos and ESCs [18]. It is essential for the maintenance of healthy development and embryogenesis [19,20,21]. SIRT1 is primarily responsible for silencing genes via heterochromatin formation. Supporting the creation of facultative heterochromatin, SIRT1 deacetylates H4K6, H3K9, and H3K56 preferentially. By acetylating and recruiting histone H1 to chromatin, SIRT1 is capable of enhancing local compactness. It is also capable of deacetylating nonhistone targets that participate in the formation of heterochromatin. SIRT1 is involved in the maintenance of cellular homeostasis during stress [22].

Accumulating evidence suggests that the deacetylation activity of SIRT1 regulates embryogenesis, development, and the maintenance of ESC pluripotency through hierarchical mechanisms [1]. SIRT1 in stem cells deacetylates histones, the epigenetic regulator DNMT3L, a critical component of the core pluripotency network OCT4, and a tumor suppressor p53 [23,24,25]. SIRT1 regulates the differentiation capacity of mouse ESCs and the expression of imprinted and germline genes [23]. Through the direct deacetylation of histones, SIRT1 inhibits the expression of differentiation-associated genes in ESCs. Reducing SIRT1 during embryonic development leads to the reactivation of those developmental genes [26]. Additionally, SIRT1 inhibits RAR-mediated activation of differentiation genes in mouse ESCs [20]. SIRT1 is essential for the maintenance of robust pluripotent ESCs as well. SIRT1 facilitates the translocation of p53 from the nucleus to the cytoplasm as a consequence of endogenous ROS damage. SIRT1 exposes ESCs to apoptosis induced by mitochondrial p53 in mice, while also impeding the suppression of Nanog expression mediated by nuclear p53 [27]. In sum, SIRT1 functions as an important regulator in coordinating metabolic and epigenetic signaling pathways to ensure the maintenance of pluripotent ESCs and normal embryogenesis through the deacetylation of key regulators.

**Figure 2:**
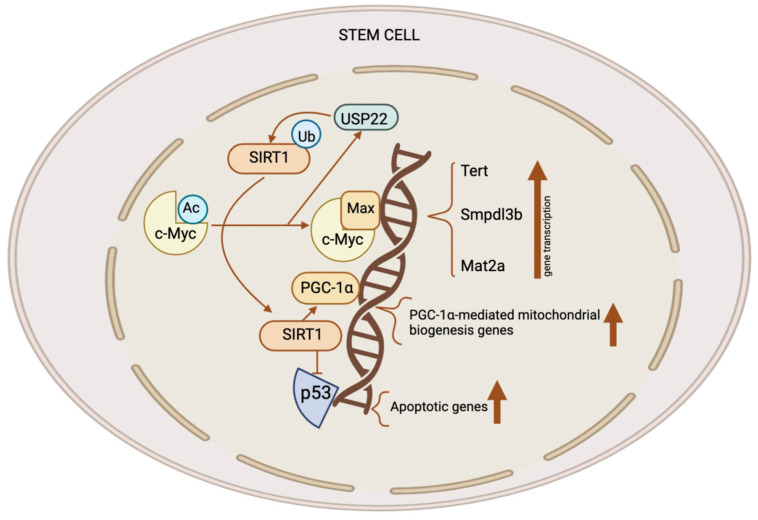
The role of the SIRT1–c-Myc axis in the control of stem cells. Both SIRT1 and c-Myc have significant expression levels in several types of stem cells. SIRT1 removes acetyl groups from c-Myc, leading to enhanced stability. When c-Myc is more stable, it increases the transcription of c-Myc target genes like Usp22, Mat2a, Tert, and Smpdl3b. The upregulation of MAT2A leads to the synthesis of SAM, resulting in an elevation of H3K4me3 levels on pluripotent genes and subsequently promoting their expression. This activity is crucial for the preservation of pluripotent stem cells. C-Myc also stimulates the production of SMPDL3B to modify sphingolipids on the plasma membrane, affecting the flexibility of the membrane and the signaling pathways which are involved in the process of neuronal development. C-Myc stimulates the process of transcribing Tert, which, in turn, enhances the elongation of telomeres. C-Myc enhances the excessive production of USP22, an enzyme which removes ubiquitin from proteins, leading to the stabilization of SIRT1. This modulation augments the suppression of p53 by SIRT1 while simultaneously increasing mitochondrial biogenesis through PGC-1α, which, in turn, promotes the survival and proliferation of stem cells. This figure was partly created with BioRender.com.

Stem cells, such as mouse ESCs and iPSCs, exhibit high expression levels of both SIRT1 and c-Myc. SIRT1 increases the stability of c-Myc in these stem cells by deacetylating it; this is likely the result of a reported exchange of K63-linked versus K48-linked polyubiquitination chains [18,28]. Enhanced c-Myc stability promotes the transcription of c-Myc target genes in stem cells, like Usp22, Mat2a, Smpdl3b, and Tert. Elevated MAT2A expression in mouse ESCs stimulates the synthesis of SAM from methionine; this, in turn, elevates the H3K4me3 content of pluripotent genes, thereby stimulating their expression [18,19]. This process is critical for the maintenance of pluripotent stem cells. Additionally, c-Myc raises the production of SMPDL3B in mouse ESCs. This changes the sphingolipids on the plasma membrane and affects signaling pathways that promote neuronal differentiation as well as the fluidity of the membrane. C-Myc increases the transcription of Tert in post-reprogrammed iPSCs in order to facilitate telomere elongation [29]. In the absence of SIRT1, iPSCs amass chromosomal aberrations and exhibit telomeric heterochromatin de-repression. Consequently, SIRT1 exerts a positive regulatory effect on TERT expression through the enhancement of c-Myc protein stability (Figure 2) [29].

SIRT2. The mitochondrial genome encodes genes essential for the mitochondrial respiratory chain, including OXPHOS subunits, rRNAs, and tRNAs. Due to proximity, mitochondrial oxidative stress may cause mtDNA mutations [30]. In fact, mtDNA mutations accumulate in stem cells with age and impede the progeny’s mitochondrial oxidative respiration.

**Figure 3:**
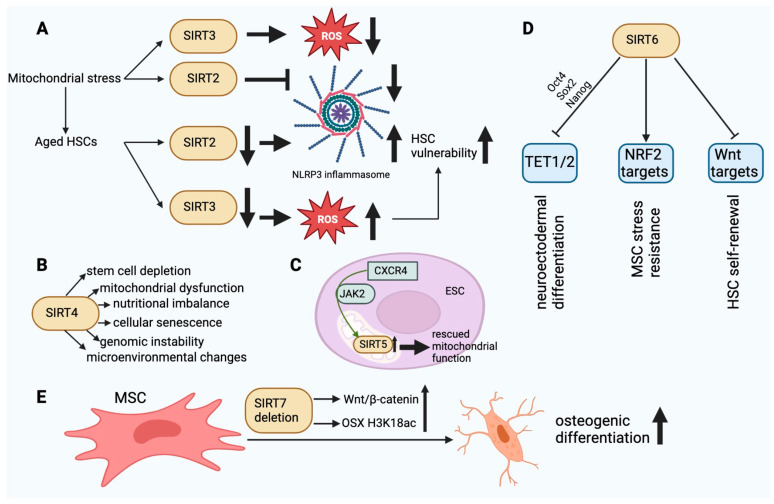
(**A**). The dysfunction of SIRT2 and SIRT3 in aged HSCs finally leads to HSC vulnerability. In aging HSCs, the activity of SIRT2 is decreased, resulting in an increase in NLRP3 inflammasome activity. In turn, the decrease in SIRT3 activity results in damage to HSCs due to ROS accumulation. (**B**). The main effects of SIRT4 in SC aging. SIRT4 dysfunction can affect the cellular functions outlined above. (**C**). SIRT5 upregulation mediated by CXCR4 via JAK2 can rescue normal mitochondrial function in ESCs. (**D**). SIRT6-mediated epigenetic regulatory functions in SCs. TET1/2 inhibition via Oct4, Sox2, and Nanog may affect neuroectodermal differentiation. Inhibition of Wnt target molecules may regulate the self-renewal capacity of HSCs. Stimulation of NRF2 target molecules may enhance the stress resistance of MSCs. (**E**). SIRT7 deletion enhances the osteogenic differentiation of MSCs in bone marrow by elevating H3K18ac levels at the promoter of the OSX transcription factor and activating the Wnt/β-catenin signaling pathway. This figure was partly created with BioRender.com.

SIRT2 is a tubulin deacetylase located in the cytoplasm that has the ability to enter the nucleus during G2–M transition. Its primary function is to regulate the cell cycle. SIRT2 is capable of deacetylating chromatin and microtubules [22]. The accumulation of mitochondrial stress leads to the activation of the SIRT2-regulated NLRP3 inflammasome [31]. SIRT2 recognizes NLRP3 as a substrate. SIRT2 deacetylates NLRP3 and thereby inhibits the activation of the NLRP3 inflammasome [32]. Aged HSCs exhibit a diminished expression of SIRT2, which leads to an abnormal activation of the NLRP3 inflammasome and a heightened vulnerability to stem cell deterioration induced by mitochondrial stress (Figure 3A) [31]. SIRT2 is able to delay glycolysis and the metabolic reprogramming of iPSCs by deacetylating four important glycolytic enzymes. These enzymes are phosphoglycerate kinase 1, enolase, aldolase, and GAPDH [33].

SIRT3. SIRT3 plays a role in mitochondrial function and metabolism. The translocation of SIRT3 to the nucleus can occur in response to calorie restriction and genotoxic stress. It can deacetylate H4K16 and H3K9 within the nucleus. SIRT3 deacetylates and activates acetyl-CoA synthetase 2 within the mitochondria, thereby augmenting the metabolic rate. In addition, SIRT3 can upregulate the expression of mitochondrial factors such as cytochrome C oxidase subunits, ATP synthetase, and transcription factor PGC1α. SIRT3 can enhance cellular survival by deacetylating Ku70, a protein which is implicated in DNA repair when exposed to genotoxic compounds [22]. In stem cells, SIRTs have been identified as pivotal regulators of the mitochondrial metabolic checkpoint [34,35]. SIRT3 functions as a deacetylase within mitochondria, modifying the antioxidant SOD2 in order to mitigate oxidative stress [36]. SOD2 expression is significantly upregulated in HSCs, whereas it is downregulated in differentiated hematopoietic cells [37]. Loss of HSC quiescence and impairment of HSC maintenance and function occur with SIRT3 deletion but not in youth. The functionality of aged HSCs can be enhanced through the overexpression of SIRT3, whereas the expression of the protein is decreased in aging HSCs [38]. Dysregulation of the mitochondrial metabolic checkpoint is an underlying cause of stem cell senescence and may be amenable to rejuvenation (Figure 3A).

SIRT4. SIRT4, an ADP-ribosyltransferase located within mitochondria, functions to impede the activity of mitochondrial glutamate dehydrogenase 1. As a result, amino acid-induced insulin secretion is decreased. By deacetylating it, SIRT4 represses the activity of malonyl-CoA decarboxylase. This blocks the oxidation of fatty acids in liver and muscle cells. The inhibitory effect of SIRT4 on PPARα reduces the rate of fatty acid oxidation. Deacetylation of ADP/ATP translocase 2 inhibits mitochondrial uncoupling, thereby increasing cellular ATP [39]. Stem cell depletion inhibits tissue regeneration and repair [40,41]. The gene products of HDAC family members facilitate the differentiation of SCs. The transcriptional downregulation of HDAC can either boost or inhibit SC self-renewal [42]. SSCs undergo dynamic changes in their transcriptional profiles throughout the processes of self-renewal, differentiation, and aging. SSCs undergoing differentiation and aging exhibits increased expression of SIRT4 and decreased expression of Hdac2, Hdac6, and SIRT1. In response to rapamycin, the expression of HDAC genes changes in SSCs, as shown by lower levels of Hdac8, Hdac9, and SIRT4 transcripts [43]. The activation of SIRT4 disrupts redox homeostasis and mitochondrial function in trophoblast SCs, which leads to the senescence of stem cells induced by lysine-specific demethylase 1 deficiency [44]. UV-induced photodamage increases the mRNA expression of SIRT1 and SIRT4 in iPSCs derived from cutaneous fibroblasts. SIRT4 undergoes degradation during the initial phase of photodamage and gradually accumulates during the subsequent phase [45]. Downregulating SIRT4 with nicotinamide mitigates DNA damage, suggesting that it could be a viable target for preventing somatic cell senescence and promoting iPSC reprogramming [46]. However, no direct evidence exists at this time to support the notion that SIRT4 is involved in iPSC reprogramming, pluripotency, or differentiation. Contrary to the aforementioned findings, the neural stem cells of adolescent mice exhibited increased SIRT4 expression in comparison to adult mice in a study. In addition, SIRT4 overexpression was found to prevent cell mortality in the presence of DNA damage, indicating that SIRT4 may serve as a protective factor to preserve the genomic integrity of neural stem cells (Figure 3B) [47].

The Wnt/β-catenin signaling pathway is essential for the development of numerous organs and the maintenance of SC self-renewal. Mechanisms of degradation and synthesis precisely regulate the maintenance of β-catenin at exceedingly low levels in the cytoplasm in the absence of Wnt ligands. Despite this, the Axin1 protein serves as the primary regulatory target throughout the β-catenin protein degradation pathway. Deacetylation of Axin1, facilitated by SIRT4 translocation from the cytoplasm to the mitochondria and by activation of the Wnt pathway signal, occurs at the K147 residue of Axin1. This deacetylation results in the accumulation of β-catenin protein and a reduction in the β-TrCP assembly of the disruption complex, both of which are consequences of the activation of the Wnt/β-catenin signaling pathway [48].

SIRT5. Cytochrome C and carbamoyl phosphate synthetase 1 are the targets of SIRT5. SIRT5 demonstrates considerable demalonylase and desuccinylase activity both in vitro and in vivo [22]. SIRT5 catalyzes the removal of negatively charged modifications, specifically malonyl, succinyl, and glutaryl groups, from the lysine residues of target enzymes, in contrast to SIRT3 and SIRT4 [49,50,51,52]. Moreover, the role of SIRT5 in metabolic regulation is contingent upon the environment, as particular metabolic processes can be either facilitated or impeded by SIRT5, depending on nutrient availability and cell type [53,54,55,56].

SIRT5 expression is downregulated in the endothelial progenitor cells of hypertensive patients, resulting in mitochondrial dysfunction characterized by impaired ultrastructure, reduced membrane potential, and increased ROS production [57,58]. It was discovered that overexpression of CXCR4 could rescue mitochondrial function in EPC-mediated angiogenesis via JAK2 signaling by upregulating SIRT5 (Figure 3C) [58]. MSCs significantly express SIRT5, which contributes to their energy requirements. On the other hand, SIRT5 levels decline considerably in MSCs that have been differentiated from adipose tissue, which is linked to higher levels of succinylation and malonylation in mitochondria [59]. Consequently, SIRT5 appears to function as an energy demand sensor in these cells. Neuronal SCs express exceptionally high levels of SIRT5, whereas it is significantly reduced in the adult brain. This indicates that SIRT5 expression is elevated in metabolically active stem cell lineages [47]. SIRT5 deficiency during culture expansion of ADMSCs leads to an abnormal metabolic pattern, increased cell proliferation, and better therapeutic effectiveness [60]. SIRT5 may be a viable target for enhancing the functional properties of MSCs in preparation for clinical application, according to these findings.

SIRT6. The function of SIRT6 has been associated with gene silencing, DNA repair, and genomic stability. SIRT6 deacetylates the histones H3K9Ac and H3K56, thereby enhancing the stability of telomeres and heterochromatin formation [22]. SIRT6 is essential for the interaction between epigenetic DNA methylation and the chromatin modifications that occur during ESC differentiation [61]. SIRT6 inhibits the expression of Oct4, Sox2, and Nanog via the deacetylation of H3K9ac and H3K56ac [62]. This inhibits the expression of Tet1 and Tet2, which regulate cell lineage selection during ESC differentiation by facilitating 5-hmC of DNA [63]. Higher amounts of 5-hmC and the overexpression of Tet1 and Tet2 cause neuronal transcription programs to become dysregulated and ESC differentiation to shift toward neuroectodermal lineages in SIRT6-deficient cells [64].

An increasing body of evidence suggests that SIRT6 plays a crucial role in the regulation of stem cell homeostasis and function across various adult stem cell types. SIRT6 is essential for HSCs’ ability to self-renew over the long term. By deacetylating H3K56, SIRT6 inhibits the transcription of genes involved in the Wnt pathway. The SIRT6 deletion decreased quiescence in HSCs [65]. SIRT6-dependent histone deacetylation plays a crucial role in MSC homeostasis. In premature or physiological aging, MSC loss may contribute to the breakdown of mesenchymal tissues. Interestingly, SIRT6-deficient rodents display degenerative defects in mesenchymal tissues [10]. When transplanted in vivo, MSCs deficient in SIRT6 demonstrate impaired differentiation of chondrocytes and osteoblasts, indications of cellular senescence, and an accelerated rate of cellular attrition [66,67]. These phenotypes are linked to oxidative stress hypersensitivity, affected redox metabolism, and the downregulation of multiple targets of the NRF2 transcription factor. It is noteworthy that MSCs exhibit a slight elevation in SIRT6 expression as they age. This may serve as a compensatory mechanism to mitigate the detrimental effects of aging-related alterations in MSC functions [67]. In a past study, SIRT6 overexpression did enhance new bone formation and repair and, by partially inhibiting NFkB signaling, improved osteogenic differentiation of rat MSCs [68]. SIRT6 plays a crucial function in preserving the epigenetic plasticity of human primary cells prior to their reprogramming into iPSCs. In cells from elderly donors, SIRT6 expression and iPSC reprogramming efficiency are reduced by more than threefold; restoring SIRT6 levels in these cells improves reprogramming effectiveness (Figure 3D) [69].

SIRT7. DNA transcription is facilitated by SIRT7 in conjunction with DNA polymerases I–III. In human cells, SIRT7 interacts with two additional molecules: RNA polymerase I and upstream binding factor [39]. Stem cell exhaustion is a combined effect of different types of damage related to aging and is likely a main cause of the breakdown of body tissues and cells [70], hindering their regenerative capacity. In SIRT7-deficient mice, there is an increase in HSCs in the bone marrow. However, these cells display a diminished capacity to repopulate the lymphoid compartment of lethally irradiated mice compared to wild-type bone marrow cells [71]. The reason for this is that the inhibition of transcription by SIRT7 of mitochondrial ribosome components is crucial in preventing HSCs from losing their quiescence, which would otherwise lead to proliferation and a decrease in survival [72,73]. In hair follicle stem cells, SIRT7 keeps telogen quiescence going. This phenomenon can be elucidated by the mechanism by which SIRT7 deacetylates residue K612 of NFATc1, thereby promoting its phosphorylation by GSK-3β, which subsequently inhibits the protein’s signaling and leads to its nuclear degradation via PA28c [74]. SIRT7 deficiency in human MSCs is associated with the emergence of various senescence-related characteristics, including retrotransposons such as LINE1 and accelerated functional wasting caused by the decondensation of heterochromatin in the nuclear periphery and activation of the cGAS-STING pathway [75]. In the context of intestinal epithelial homeostasis, SIRT7 plays a critical role, particularly in the maintenance of LGR5+ stem cells which are essential for epithelial regeneration. A deficiency in this protein results in aberrant chromosome segregation, an insufficient response to DNA damage, and insufficient intestinal cell differentiation. Furthermore, it increases Wnt signaling and the expression of genes associated with colorectal cancer [76]. MiR-152 has been identified to increase with the aging of human DPSCs and to promote the senescent phenotype via its regulatory effect on SIRT7 levels [77]. By increasing the H3K18ac levels at the promoter of the OSX transcription factor and by activating the Wnt/β-catenin signaling pathway, SIRT7 deletion promotes the osteogenic differentiation of mesenchymal stem cells in bone marrow (Figure 3E) [78,79]. This is particularly true for isoforms 1 and 2. RBM6 guides the binding of SIRT7 to the OSX promoter, which is governed by the lncRNA PLXDC2-OT [79]. Additionally, a protracted in vitro culture of iPSCs resulted in the detection of low levels of SIRT7. These results replicate various aspects related to premature aging syndromes and somatic cell senescence, as documented by previous studies [80]. SIRT7-mediated maintenance of stem cell quiescence is critical for population preservation. However, it can disrupt processes which require stem cells to differentiate, necessitating appropriate regulation. Exosomes secreted by bone marrow MSCs carrying miR-125b downregulate SIRT7, enhancing the response to myocardial ischemia-reperfusion injury [81]. This may be achieved by facilitating the replacement of damaged cells in a more timely and effective manner. In the same way, exosomal miR-17-5p, which comes from human umbilical cord mesenchymal stem cells, lowers SIRT7 to improve ovarian function in women who have already lost their ovaries [82]. Overexpression of SIRT7 in mouse embryonic fibroblasts has been shown to inhibit cell proliferation and growth by activating transcription in the absence of p53 and c-Myc [83].

In conclusion, it is evident that SIRTs serve a critical regulatory function in SCs. The SIRT family is known for its roles in metabolic diseases and aging. They are also important regulators of SC biology, acting as stress and metabolic sensors in cells. Diverse SIRTs exhibit cell type-specific and/or stage-dependent effects on stem cells in response to environmental stimuli, regardless of their shared reliance on intracellular NAD+. Nuclear and cytosolic SIRTs have an effect on pluripotent stem cells through epigenetics, redox homeostasis, controlling metabolism and the stress response of cells, and managing the factors that determine pluripotency. SIRTs safeguard against the stress-induced and aging-induced depletion of adult stem cells by preserving their capacity for self-renewal, quiescence, and regeneration.

Regarding carcinogenesis, SIRT1, SIRT3, SIRT4, and SIRT6 operate similarly in healthy cells to tumor suppressor proteins. They independently advocate for the maintenance of genomic stability and DDR. SIRT3 and SIRT6 are conceivably the finest examples of how numerous SIRTs activate “real” tumor suppressor proteins, such as gatekeepers and caretakers, while inactivating oncoproteins. In these particular circumstances, the mere presence of upregulated SIRTs in certain cancer cells does not necessarily imply that they play a causal role in the development of cancer [84]. However, impaired SIRT functions can lead to DNA damage, genomic instability, and chromosomal aberrations, which can be the basis of tumorigenesis [84].

## 3. SIRTs and Autophagy

Macroautophagy is a highly conserved lysosomal degradation mechanism [85]. In contrast to microautophagy and CMA, macroautophagy is characterized by the creation of autophagosomes, which are double-membrane vesicles. These vesicles not only encapsulate intracellular constituents, including proteins, macromolecular complexes, and organelles, but also invading pathogens [86]. Macroautophagy occurs at basal levels in virtually all eukaryotic cells. However, a wide range of pathological and physiological stimuli often induces macroautophagy due to cellular stress.

Protein acetylation has been shown to be a key regulatory mechanism of autophagy. SIRTs have been implicated in the acetylation of many autophagy-associated proteins [87,88,89,90,91,92,93,94,95,96]. SIRTs have an effect on autophagy initiation, the MAP1LC3/LC3 conjugation system, and the control of autophagy-associated genes [97].

Induction of autophagy takes place upon the activation of ULK1 in response to starvation [98]. ULK1 regulates autophagy initiation by forming a protein complex with multiple partners, including RB1CC1/FIP200, ATG13, and ATG101 [99,100,101,102,103]. AMPK and mTORC1 regulate the activity of the ULK1 complex [104]. The ULK1 complex activates the BECN1-PIK3C3/VPS34 complex, facilitating the local synthesis of PtdIns3P [105]. The recruitment of the downstream effector WIPI2 by PtdIns3P promotes the expansion and formation of phagophores [105]. SIRT1 serves as the principal deacetylase for the K437 and K430 of BECN1 [106]. In addition to autophagy, BECN1 and PIK3C3 collaborate in the formation of numerous protein complexes that are involved in other membrane processes [107,108,109,110]. Thus, the EP300-mediated acetylation of PIK3C3 and BECN1 and the SIRT1-mediated deacetylation of BECN1 could affect a variety of cellular membrane processes.

As a tumor suppressor and participant in autophagy induction, FoxO1 is deacetylated by SIRT2 in H1299 NSCLC cells [111,112]. SIRT2 inhibition increases FoxO1 acetylation and facilitates the interaction between FoxO1 and Atg7, which is necessary for the initiation of autophagy [111]. Colon or lung cancer cells exhibit this effect [112]. Overexpression of SIRT2 has been closely associated with the induction of the protective autophagy mechanism of HL-60/A cells [113].

**Figure 4:**
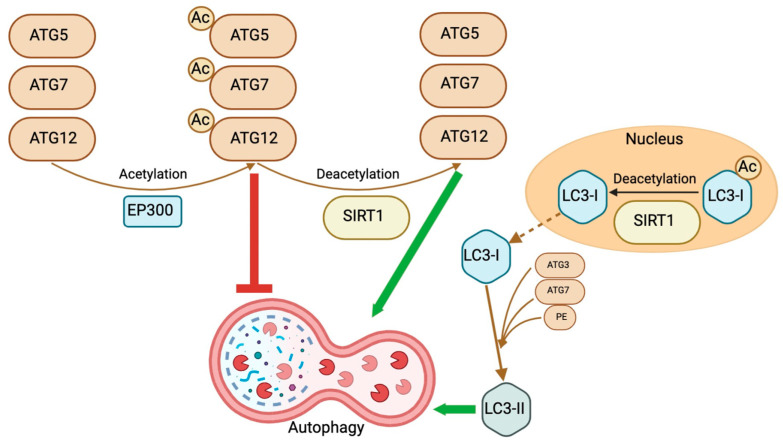
Schematic representation of the role of SIRT1 in autophagy. EP300-mediated acetylation of ATG5, -7, and 12 molecules causes the inhibition of autophagy. Conversely, SIRT1-mediated deacetylation of these molecules stimulates autophagy. Deacetylation of LC3-I promotes translocation to the cytoplasm and then, via ATG3, ATG7, and PE, the formation of LC3-II, stimulating the mechanism of autophagy. This figure was partly created with BioRender.com.

A crucial event in autophagy is the transformation of soluble LC3 into membrane-bound LC3-PE. Membrane-attached LC3-PE regulates several crucial autophagy processes, including phagophore growth and expansion, cargo recruitment, and lysosome fusion [114,115,116]. The process of LC3-PE formation is dependent on the ubiquitination-like conjugation system, in which soluble LC3 becomes bound with PE with the aid of the E3-like ATG12–ATG5–ATG16L1 complex, the E2-like enzyme ATG3, and the E1-like enzyme ATG7 [97,105,106,117,118]. SIRT1 functions as the principal deacetylase, eliminating the acetyl group from lysine residues ATG5, ATG7, and LC3 that have been acetylated (Figure 4) [87].

The regulation of autophagy occurs via transcriptional mechanisms [119,120]. TFEB, a member of the MiT/TFE family, upregulates the expression lysosome and autophagy-associated genes such as MAP1LC3B, SQSTM1, LAMP1, and cathepsin D [120]. Multiple protein kinases, including mTORC1, regulate TFEB [121,122,123]. The phosphorylation of TFEB by mTORC1 inhibits its transcriptional activity and impedes its translocation into the nucleus [123]. The transcription of genes associated with autophagy is also intricately connected to the acetylation of histones [124,125]. KAT5/TIP60 acetyltransferase facilitates the activation of autophagy-related gene transcription, while KAT8/MOF induces repression. SIRT1 reverses the KAT8/MOF-mediated acetylation of histone H4K16, which represses the transcription of several autophagy-related genes (e.g., NBR1, MAP1LC3B, and ULK1) [124].

Directing the acetylation states of proteins associated with autophagy are signaling molecules in response to cellular nutrients and growth factors. An AMPK-dependent mechanism triggers SIRT1 in the absence of glucose [126]. The nuclear translocation of GAPDH is induced by AMPK-mediated phosphorylation [126]. By displacing cell cycle and apoptosis regulator 2 (AR2)/Deleted in breast cancer-1 (DBC1), the principal repressor protein of SIRT1, nuclear GAPDH, subsequently binds and activates SIRT1 [126,127,128]. SIRT1 is then activated and deacetylates nuclear LC3 to promote autophagy initiation [87,88,126]. It is unknown, however, whether the deacetylation of additional ATG proteins by SIRT1 also involves nuclear events.

A conserved process, microautophagy involves the direct ingestion and degradation of cytosolic components by endolysosomal compartments in mammals or the vacuole in yeast. Cargoes of microautophagy consist of organelles and proteins, including peroxisomes, mitochondria, and nucleus fragments [129,130,131,132]. There are two distinct mechanisms of lysosomal cargo sequestration in mammalian cells: lysosomal invagination and protrusions of the lysosome’s limiting membrane [132]. SIRTs are also involved in the regulation of certain types of microautophagy [133].

CircRNA is a large group of non-coding RNAs that have a closed-loop structure and may act as miRNA sponges, providing binding sites for miRNAs which control the expression of the target gene [134]. Increasing evidence suggests that certain circRNAs, including circErcc2, may function as miRNA inhibitors [128]. As a direct target of circErcc2, mir182-5p inhibits the expression of SIRT1, which, in turn, stimulates apoptosis and inhibits mitophagy. As a result, in response to oxidative stress, overexpression of circErcc2 could substantially inhibit apoptosis and ECM degradation, while increasing mitophagy via targeting Mir182-5p-SIRT1 [135].

Specific cell types exhibit a comparable correlation between mitophagy and oxidative stress. SIRT2 targets PPARGC1A/PGC-1A in order to inhibit mitophagy and protect annulus fibrosus cells from apoptosis in the presence of oxidative stress [136]. This shows that PPARGC1A is linked to mitophagy in annulus fibrosus cells and probably in other cell types where the quality control of mitochondria is important [133].

Honokiol, a small-molecule organic compound agonist of SIRT3 [137], increases the lipidation of the marker for autophagic vacuoles, LC3, and upregulates the expression of two mitophagic markers, BNIP3 and BNIP3L. Furthermore, nucleus pulposus cells treated with honokiol contain a greater number of mitophagic vacuoles and a greater colocalization of LC3 and BNIP3L compared to untreated cells. SIRT3 inhibition, on the other hand, eliminates these honokiol-induced mitophagy-associated changes. This proves that honokiol promotes initial mitophagy via SIRT3 [138].

Lipophagy, akin to bulk nonselective autophagy, can transpire through microautophagic or macroautophagic pathways, contingent upon how lipid droplets are conveyed into lysosomes or vacuoles [139,140].

Lipid droplets can be selectively targeted for macrolipophagy in an Ub-independent manner. SIRT1 is required for PNPLA2-mediated macrolipophagy [87,141]. In primary hepatocytes, SIRT1 ablation prevents the upregulation of Atg gene expression, macroautophagic flux, lipophagy, and TAG turnover resulting from PNPLA2 overexpression. The precise mechanism through which PNPLA2 controls SIRT1 remains a subject of ongoing research [141]. Macrolipophagy is also coordinated by the main cellular energy-sensing pathways that regulate autophagy. This includes the well-known nutrient-responsive and stress pathways that are downstream of mTORC1 and PRKA. Inhibiting mTORC1 facilitates the autophagy-dependent reduction in lipid accumulation in vivo [142]. On the other hand, PRKA has the ability to stimulate autophagy and macrolipophagy via multiple mechanisms, including the activation of SIRT1, the stimulation of ULK1 [104], and the inhibition of mTORC1 [143,144].

In addition to the above mechanisms, there are a number of less well-understood ways in which SIRTs may be involved in the regulation of different forms of autophagy. SIRTs regulate macroautophagy primarily through the AMPK/mTOR signaling pathway. In addition, SIRT1, SIRT3, SIRT4, and SIRT5 are implicated in Bnip3-mediated or PINK1/Parkin-mediated mitophagy [145].

The regulatory functions of acetyltransferases and deacetylases in macroautophagy have been the subject of extensive research. However, their involvement in alternative forms of autophagy remains inadequately comprehended. The deacetylation of mitochondrial proteins initiates mitophagy, indicating that protein acetylation plays a role in regulating selective autophagy. Nevertheless, the precise mechanisms by which protein acetylation-based signaling pathways regulate selective autophagy remain mainly unknown.

## 4. SIRT Regulation of Autophagy in Cancer Stem Cells

Previously, the important role of SIRT proteins in stem cell biology and autophagy regulation has been discussed. Unregulated cellular division distinguishes cancer, which progresses through the accumulation of epigenetic and genetic alterations. A number of investigations have been undertaken over the last ten years to examine the involvement of SIRTs in the development of cancer [145,146]. Frequently, SIRTs play a dual role in the progression of cancer, functioning as both tumor suppressors and promoters. This contradictory function likely varies according to the cancer type under consideration and the distinct signaling pathways that become active during the progression of the disease.

The two-faceted relationship between cancer and autophagy is well known, presenting aspects which have been discussed from different angles in several of our articles [147,148,149,150,151,152]. Mammalian cells exploit regulatory processes between SIRTs and autophagy to maintain energetic homeostasis in response to internal and external stimuli. There are several interfaces among SIRTs and autophagy that modulate homeostatic mechanisms. More details are already known about the role of different SIRTs in the function of cancer cells through autophagy and their changes in different cancer types [153]. The same players and mechanisms can harness the accumulation of mutations or failure to cope with stressors in cells for the benefit of cancer. Here, we discuss aspects of SIRT-mediated autophagy regulation from the perspective of cancer stem cells.

Heat-stable antigen CD24, homing cell adhesion molecule CD44, CD90 (Thy1), CD133 (prominin-1), CD200 (OX-2 membrane glycoprotein), epithelial-specific marker (EpCAM), and nestin have been utilized thus far to identify CSCs [154]. The CSCs hypothesis posits that CSCs constitute a substantial impediment to the development of effective cancer therapies. This can be an explanation for the therapeutic tolerances and survival behaviors displayed in tumors. Aberrant epigenetic modifications have the potential to facilitate the expression changes of CSC markers in tumor cells, thereby promoting the formation of a diverse population of tumor cells [155]. As a result, epigenetic regulation of CSCs may offer a novel therapeutic approach to the treatment of cancer.

SIRTs modulate multiple pathways involved in the maintenance of stem cell functions. Hence, SIRTs display a pivotal role in the development of organisms and in tissue homeostasis. The Wnt, Notch, and Hedgehog signaling pathways are essential for determining the properties of stem cells, including self-renewal and differentiation [156]. A number of epigenetic mechanisms, including methylation and acetylation, rigorously control these pathways [156]. These modifications are facilitated by SIRTs, specifically SIRT1. Within this particular framework, CSCs delineate a compact assemblage of cells that possess the ability to regenerate themselves and generate diverse cell types, thereby forming a tumor. CSCs exhibit aberrant signaling pathways that may result in symmetrical or asymmetrical self-renewal of the cancer stem cell pool or tumor differentiation. SIRT1 has been extensively examined in CSCs, as in normal cells, with a particular emphasis on its high expression in glioma, breast cancer, colorectal cancer, and leukemia [157,158,159].

Regarding tumorigenesis, SIRT1 generates controversy due to its ability to deacetylate oncogenes including β-catenin, survivin, and NFkB as well as tumor suppressors including p53 and the FoxO transcription factor [160]. SIRT1 has been shown to act as a crucial promoter in the maintenance and self-renewal properties of cancer stem cells (e.g., glioma, colorectal, breast, pancreatic CSCs, CML, and AML SCs) [161,162,163,164,165]. These SCs highly express SIRT1. SIRT1 is an intricate component of PRC4 [166]. It was reported that parts of PRC4, like EZH2, Suz12, Eed, and SIRT1, turned up in different kinds of cancerous tissue, like breast and prostate cancer. SIRT1 has been found to be directly associated with Suz12 in CSCs. Furthermore, SIRT1 and polycomb-group proteins are overexpressed in breast and colon malignancies [166]. The onco-fusion protein BCR-ABL, which is produced in CML through the activation of SIRT1 mediated by STAT5, is essential for both the malignant transformation and the development of hematopoietic progenitor cells. Through the deacetylation of different substrates, such as FoxO1, P53, and Ku70, SIRT1 helps cancer grow and ensures cell survival. In this way, inhibiting SIRT1 could potentially decrease the survival and proliferation of CML SCs while increasing their sensitivity to TKI treatments [167,168].

SIRT1 was discovered to be highly expressed in glioma cells positive for CD133. This protein is important in tumor recovery and resistance to radiotherapy. In vitro experiments demonstrated that inhibiting SIRT1 expression in these cells resulted in increased radiosensitivity and apoptosis caused by radiation. Furthermore, SIRT1 suppression substantially boosted the mean survival rate of mice undergoing radiotherapy and harboring glioblastoma-CD133-positive tumors [169]. The expression of SIRT1 is increased in CNSCs and GSCs (those positive for CD133, Sox2, and nestin). SIRT1 was found to be crucial in evading p53-dependent tumor surveillance; thus, it induced oncogenic transformation and preserved the neural cancer stemness of the cells [161]. Furthermore, SIRT1 expression was found to be reduced in glial cells (GFAP-positive) after differentiation from GSCs [161]. In addition, translation of SIRT1 was inhibited in nestin and NICD-positive GSCs by CPEB1, a key modulator which induced differentiation at the post-transcriptional level, according to another study [170]. The aforementioned evidence suggests that SIRT1 and CPEB1-mediated translational control are crucial differentiation factors in GSCs and might serve as a viable therapeutic target for GSCs.

There is an existing molecular link between CD133 and autophagy. Src family tyrosine kinases phosphorylate the intracellular C-terminal domain of CD133. This allows the p85 subunit of PI3K to bind and become activated. Subsequently, PI3K activation instructs downstream targets, including Akt, to stimulate cellular proliferation. CD133 is additionally stabilized through its binding to HDAC6, an enzyme which promotes cell proliferation, inhibits cell differentiation, and enhances the transcriptional activity of β-catenin [171]. Unphosphorylated CD133 is transported from the plasma membrane to intracellular regions via endocytosis when Src family kinases are inactive. Dynein and HDAC6 motors facilitate the transport of CD133 endosomes to centrosomes via microtubules upon the completion of endocytosis. CD133 localizes endosomally to the centrosome, where it binds GABARAP and impedes the interaction between GABARAP and ULK1 as well as the initiation of autophagy [171].

Autophagy and Sox2 also display cross-talk. Sox2 induces autophagic events, such as the formation of intracellular vacuoles and the activation of lysosomes in colon cancer cells [172]. Sox2-mediated expression of the ATG10 gene activates autophagy, suppressing ex vivo cell proliferation, anchorage-independent colony growth, and in vivo tumor growth. Furthermore, Sox2 induces the upregulation of tumor suppressors or senescence factors, such as phosphorylated p53, p16, and p21, thereby augmenting cellular senescence. Notably, knocking down ATG10 restores cancer cell properties in Sox2-expressing colon cancer cells [172].

Also, experimental data strongly suggest that there is a complex regulatory link between nestin and SIRT1-regulated autophagy [173]. In post-ischemic neurogenesis, nestin and LC3 isoforms coexist inside viable cells. This suggests the involvement of the SIRT1/autophagy pathway in their activation.

It was observed that CD24-negative breast CSCs contained a high level of SIRT1, while CD44-positive CSCs contained a low level of miR-34a. Subsequent investigations revealed that miR-34a partially inhibited the proliferative potential of breast CSCs and reduced the expression of CSC markers ALDH1, polycomb ring finger proto-oncogene Bmi1, and homeobox transcription factor Nanog by downregulating SIRT1 [174]. This mechanism may also be present in pancreatic CSCs [163]. In colorectal CSCs and neuronal SCs, miR-34 is also implicated in autophagy modulation, partly via the Wnt–autophagy axis [174], and it also regulates EMT [175]. Further, the function of CD44 bypasses autophagy dysfunction and aging [176].

Additionally, in another study, a significant number of CRC specimens exhibited elevated SIRT1 expression, which was clearly associated with a poor prognosis for CRC patients. Furthermore, SIRT1 has been found to be highly expressed in stem-like colorectal cancer cells, its expression co-localized with CD133, a widely used marker of colorectal CSCs. In vivo, SIRT1 inhibition inhibits tumorigenicity, dampens colony and sphere formation in vitro, and decreases the proportion of CD133-positive cells. In the absence of SIRT1, stemness-associated gene expressions of Oct4, Cripto-1, Nanog, TERT, and Lin28 are downregulated [162]. Further, Oct4, Cripto-1, Nanog, TERT, and Lin28 are all involved in autophagy-regulating networks [177,178,179,180,181]. These findings suggest that the interaction of SIRT1 and autophagy is essential for maintaining the stem-like properties of CRC cells. The SIRT1/autophagy interplay may serve as a prognostic indicator of tumor recovery risk in cancer patients and could help in the development of a novel therapeutic approach to cancer treatment.

As shown above, the SIRT1–CSC marker–autophagy axis is a complex, intricate regulatory network that may have profound biological implications for CSCs.

SIRT1 is overexpressed in human LSCs as well. Inhibiting it has been found to increase leukemia SC apoptosis and inhibit CML SC or primitive progenitor cells’ proliferation. In addition, imatinib, a BCR-ABL TKI, facilitates the elimination of CML SCs by inhibiting SIRT1 in part via p53 activation [164]. SIRT1 homozygous mutant BALB/c mice exhibited a reduction in CD150-negative side-population CML stem cells and compromised their maintenance. Knocking out SIRT1 inhibits CDK6 expression, while the deacetylates of p53 activates it [182]. With the exception of CML stem cells, primary human FLT3-ITD AML stem cells also exhibit high levels of selectively expressed SIRT1. The proto-oncogene c-Myc further enhances this expression by inducing elevated levels of USP22, a deubiquitinase. Additionally, the co-administration of FLT3 and SIRT1 inhibitors leads to a decrease in FLT3-ITD AML stem cells [165]. It may be possible to treat BCR-ABL CML and FLT3-ITD AML more effectively by blocking the SIRT1 network along with TKIs or FLT3 inhibitors [183].

Significant levels of SIRT1 and c-Myc expression have been identified in stem cells, including leukemia stem cells [184]. C-Myc induces the post-transcriptional upregulation of USP22, which stabilizes SIRT1 in LSCs [165]. This control promotes LSC survival and proliferation by enhancing the SIRT1-mediated inhibition of p53 and stimulating PGC-1α-mediated mitochondrial biogenesis.

**Figure 5:**
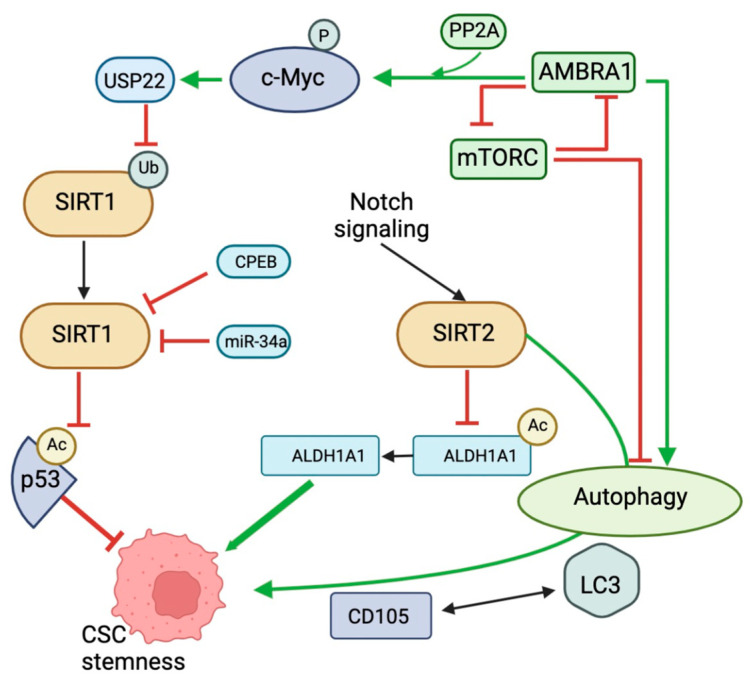
Schematic overview of several molecular cross-talk between SIRT1/2 and autophagy in CSCs. SIRT1 affects acetylated p53, which regulates the stem cell phenotype of CSCs. SIRT1 function is also affected by miR-34a, CPEB, and c-Myc. c-Myc may be stimulated by mTORC–AMBRA1 interaction via PP2A, thereby indirectly affecting SIRT1 and CSC function. Notch signaling regulates SIRT2 function, which may affect CSC function by inhibiting the deacetylation of ALDH1A1. However, SIRT2 also affects autophagy and may influence changes in the phenotype of CSCs mainly through the LC3–CD105 interaction. This figure was partly created with BioRender.com.

The deacetylation of c-Myc by SIRT1 enhances its affinity for Max [185,186]. SIRT1 also promotes the deacetylation of c-Myc, which increases K63-linked polyubiquitination and inhibits degradative K48-linked polyubiquitination. This increases p300 recruitment and c-Myc stability [187]. Both mechanisms facilitate the transactivation of c-Myc target genes, such as those involved in metabolism, cell cycle, and proliferation. On the contrary, c-Myc also promotes SIRT1 activity [188,189]. The NAMPT enzyme is responsible for limiting the rate of the amidated NAD+ salvage pathway. C-Myc increases cellular NAD+ initially through the transcriptional activation of NAMPT. Increased NAD+ levels stimulate SIRT1’s deacetylase activity. Furthermore, c-MYC potentially enhances the activation of SIRT1 by directly binding to the SIRT1 inhibitor Deleted in Breast Cancer 1 (DBC1) and blocking its interaction with SIRT1. In p53-deficient cells, c-Myc ultimately stimulates SIRT transcription in a direct manner (Figure 5) [188,189].

SIRT1 and c-Myc may establish a positive feedback loop in cancer cells. When c-Myc is activated, SIRT1’s activity and expression rise, which deacetylates c-Myc. The deacetylation of c-Myc enhances both the transactivation activity and stability of the protein. The positive feedback loop between SIRT1 and c-Myc supports cells to respond properly to both endogenous and external stimuli [19]. The function of c-Myc is also widely involved in and regulated by autophagy-related mechanisms [190]. Previous research has established that AMBRA1 is involved in embryonic stem cells during both autophagic pro-survival response and Beclin-1-dependent autophagy [191]. AMBRA1 maintains the equilibrium between autophagy and apoptosis in colorectal cancer cells, according to research [192]. AMBRA1 is an essential regulator of these cellular processes. AMBRA1 facilitates the degradation and dephosphorylation of c-Myc, as well as the interaction between c-Myc and PP2A. This decreases the rate of cell division [193]. AMBRA1 has been identified as a target of mTOR in the autophagy pathway [194]. In addition, mTOR controls the AMBRA1/PP2A-mediated regulation of c-Myc [193], demonstrating that mTOR plays a crucial role in cellular fate regulation by interfering with the metabolic state of c-Myc.

SIRT2 has been implicated in the carcinogenesis and early lineage commitment of ESCs [195,196]. SIRT2 can be induced by Notch signaling, resulting in ALDH1A1 deacetylation and enzymatic activation, which promote breast CSCs (Figure 5) [197]. Resveratrol has been shown to have a dual effect on GSCs: it inhibits cell cycling at low concentrations and induces necrosis at high concentrations, while sparing healthy human neural SCs. SIRT2 activity mediates the inhibitory effect of resveratrol on the GSC cell cycle, as revealed by the pharmacological inhibition of SIRT2 and the downregulation of SIRT2 expression with silencing RNAs. Conversely, higher concentrations of resveratrol induce GSC necrosis, which is not influenced by SIRT activity [198]. Additionally, skin malignancies rather than normal skin exhibit an upregulation of the stem cell marker CD34 upon SIRT2 deletion [199,200]. In addition to CD133, CD105, and CD44, SIRT2 has been identified as a potential unique marker for renal cell CSCs [201,202]. Due to the notable inverse relationship between LC3 and CD105 expression [203] and the established impact of SIRT2 on autophagy [204], it is plausible that SIRT2 may also affect the viability of renal cell CSCs through CD105.

A recent study examined the expression of SIRTs in individuals with lymphoproliferative diseases who were undergoing CD34+ cell mobilization [205]. There was a notable rise in the expression levels of all SIRTs except SIRT4 on the day after the first apheresis in comparison to the day prior to mobilization. The administration of chemotherapy during mobilization was linked to the heightened expression of these SIRTs. Chemotherapy disrupts the ecological conditions of the bone marrow habitat and induces hematopoiesis in response to stress. Chemotherapy medicines operate as stressors that harm both progenitor cells and their offspring while also promoting the growth of HSCs. The stress caused by chemotherapy triggers oxidative phosphorylation in the mitochondria [206]. The notable rise in the expression of SIRTs, specifically SIRT3 and SIRT5, can be attributed to this phenomenon. The involvement of SIRT3 is particularly significant since it controls the acetylation patterns of mitochondrial proteins during the oxidative stress that follows chemotherapy [207]. Furthermore, the upregulation of SIRT3 is mostly detected in youthful HSCs, which may be found in the peripheral blood after mobilization and apheresis [37]. One cannot exclude the possibility that the stem cell-protective effects of SIRT3 and SIRT5 could be mediated by different forms of autophagy [208,209,210,211,212,213].

SIRT4 has a negative regulatory effect on SIRT1 expression by suppressing glutamine metabolism [214]. SIRT4 targets H4K16ac and BRCA1 as new key factors in breast cancer cells and breast CSCs [214]. Previously, we discussed the strong association between SIRT1 and autophagy. SIRT4 potentially controls autophagy by modifying SIRT1 expression. Additionally, SIRT4 can directly impact the autophagy mechanism itself [215,216,217]. 

The overexpression of SIRT6 hampers the proliferation of CSCs and the formation of tumors by disrupting the PI3K pathway, which is a mechanism which is not connected to its deacetylation activity [218]. According to reports, primary ESCC samples exhibited elevated levels of SIRT6 expression [219]. SIRT6 suppressed the activity of mTOR and promoted the process of LC3B-mediated autophagic flux in ESCC cells by interacting with ULK1. SIRT6 concurrently promoted cellular proliferation and played a role in regulating the G2M phase. These data suggest that SIRT6 may promote cancer and initiate autophagy.

At its core, SIRTs could be very important in managing the number of CSCs because they manage how cells react to different types of stress, including hypoxia, autophagy, and mitophagy [153].

In sum, SIRTs regulate critical nodes of CSCs and carcinogenesis to such an extent that the distinction between cancer promotion and cancer suppression is extremely delicate and should be evaluated on a case-by-case basis. SIRTs can definitively regulate autophagy and mitophagy in CSCs by inhibiting transcription factors or proteins that are components of the autophagy and mitophagy machinery. This is an additional emergent aspect of significance. SIRTs are also capable of regulating autophagy and mitophagy via modulation of the CSC metabolism. This demonstrates how a comprehensive understanding of the function of each SIRT component in a specific cancer cell pathway could be crucial for the development of novel anti-tumor strategies, either independently or in conjunction with existing therapies.

## 5. Future Perspectives

Recent data indicate that SIRTs may play a crucial role in the intersection of stemness, autophagy, and cancer. To effectively fight CSCs and their tumor-promoting activities in the future, it is likely that treatments that target multiple factors at the same time will be combined. This is because the complex and variable relationship between SIRTs and autophagy is still not well understood. The majority of research adopts a focused methodology to investigate the mechanisms via which SIRTs control autophagy. They have directed their efforts towards investigating the mechanisms by which SIRTs control the signaling pathways connected with autophagy, previously implicated in CSCs. Alternatively, they have also sought to determine the extent to which the established roles of SIRTs in regulating autophagy are influential in CSCs. The most suitable strategy in this regard would be to utilize impartial, high-throughput experimental methods since they have the potential to offer a more detailed understanding of the underlying mechanisms. Proteomics, specifically high-resolution mass spectrometry-based proteomics, has previously been used to discover proteins and substrates that interact with SIRTs in the process of autophagy [4,220]. It is possible to propose that employing comparable methods on stem cells or cancer stem cells may uncover new roles for SIRTs and autophagy. An in-depth analysis of target autophagy-related genes regulated by SIRTs in specific subcellular populations could provide valuable insights and contribute to the existing research on transcriptome analysis, DNA methylome, and histone modifications in CSCs [156,220].

Therapeutic manipulation of the relationship between SIRTs and autophagy may open new avenues in cancer therapy. There is little evidence yet that SIRTs have an effect on immunotherapy, but we do know that SIRTs play an important role in immunity [221]. Several SIRTs, such as SIRT1, -2, and -6, may regulate NFkB-driven immune response through the deacetylation of proteins. Based on recently published results, we know that SIRT7 inhibits PD-L1 expression in HCC cells. SIRT7 achieves this by reducing the acetylation of MEF2D [222]. Modulating the activity of SIRT7 may improve the efficacy of immunotherapies against HCC. SIRT modulators, both activators and inhibitors, may have anti-tumor effects. Resveratrol and piceatannol may raise the level of PD-L1 in breast and colorectal cancer cells by activating SIRT. This may happen through HDAC3/p300-mediated NF-κB signaling. SIRT activators, along with anti-PD-L1 immunotherapy, may be an effective therapeutic option for cancer patients with low PD-L1 expression [223].

The SIRT1 activator resveratrol was found to induce protective autophagy in non-small-cell lung cancer through inhibiting the Akt/mTOR and activating the p38-MAPK pathways [224]. As a result, autophagy inhibition may augment the anti-tumor properties of resveratrol in this type of cancer. UBCS039 has demonstrated efficacy as a SIRT6 activator. UBCS039 has been shown to induce autophagy-dependent cell death in human colorectal cancer H1299, human fibrosarcoma HT1080, human colon cancer HCT116, and human epithelial cervix carcinoma HeLa cells [225].

Epigenetic alterations of autophagy in aggressive cancers can intentionally impact immunosurveillance, maintenance, resistance to treatment, and invasion. Hence, comprehending the fundamental mechanisms implicated in the epigenetic control of autophagy might augment the cytotoxic impact of treatments, thereby eradicating resistance in tumor cells and averting illness recurrence. Furthermore, the utilization of epigenetic modulators, such as demethylating drugs or HDAC inhibitors, not only seeks to restore abnormal epigenetic patterns on DNA sequences or histones but also presents a novel therapeutic possibility for controlling autophagy in cancer cells and CSCs [226,227,228].

## 6. Conclusions

Although SIRT1 and SIRT2 have been extensively studied in relation to autophagy and CSCs, new findings have emerged for other SIRTs. SIRT1 translational control is essential for CD133+ glioma stem cell development. CD133 activates autophagy via GABARAP and ULK1 [161,169,170,171]. In CRC stem cells, SIRT1–Sox2 (and p53, p16, and p21)–ATG10 molecular cross-talk inhibits cell growth [172]. The fact that SIRT1–nestin–LC3 molecules interact with neurogenesis and stem cells supports their significance [173]. SIRT1–CD44–miR-34a interactions have been shown to inhibit breast cancer stem cell growth and downregulate SIRT1. MiR-34a regulates the Wnt–autophagy axis, promoting epithelial-to-mesenchymal transition in CRC CSCs and neural SCs [163,174,175,176]. SIRT1, autophagy, and CRC CSC markers (CD133, Oct4, Cripto1, Nanog, TERT, and Lin28) are all connected. These molecular linkages affect CRC development and recurrence [162,177,178,179,180,181]. BCR-ABL TKI suppresses SIRT1 via p53 in leukemia stem cells, whereas c-Myc upregulates it. The C-Myc–AMBRA1 molecular connection could promote ESCs and CRC CSCs survival by activating Beclin1-dependent autophagy. The autophagy mechanism relies on the mTOR-dependent regulation of AMBRA1/PPA2-mediated c-Myc activity [164,165,183,184,193,194].

SIRT2 promotes breast cancer CSCs via Notch–autophagy connections and ALDHA1A deacetylation [197]. Renal cell CSCs also have SIRT2–CD105–LC3 molecular cross-talk, which affects their survival [203,204].

After HSC transplantation, SIRT3, SIRT5, and CD34 may impact autophagy to protect stem cells. SIRT4 might affect breast cancer CSCs by altering SIRT1 expression, targeting H4K16Ac and BRCA1, and affecting the process of autophagy [205,206,207]. SIRT6 increases the LC3B-mediated autophagy flux in ESCCs, promoting cell proliferation [218,219].

Although more and more details are emerging about the role of SIRT-mediated autophagy in CSCs, there are still many gaps in our knowledge in this area. There are many unanswered questions, such as the following: (1) what is the exact role of SIRTs in CSC metabolic pathways, and how does this affect macroautophagy, microautophagy, and selective autophagy types? (2) The molecular mechanisms by which SIRTs interact with autophagy regulatory proteins are also not known in detail. Nor do we know exactly how autophagy affects the function of SIRTs. (3) Understanding how SIRTs can keep cancer stem cells dormant or destroy them holds enormous therapeutic potential. This could eliminate the basis for tumor recurrence. (4) Is there one, and if so, what are the therapeutic implications of knowing the exact status of SIRTs and autophagy in each cancer?

Unraveling the molecular relationships that are still unknown today and understanding their biological significance is essential for developing new therapeutic tools against cancer. 

## Data Availability

No new data were created or analyzed in this study. Data sharing is not applicable to this article.

## Abbreviations

5-hmC: 5-hydroxymethylcytosine; ADMSC: adipose-derived MSC; ADP: adenosine diphosphate; ALDH1: aldehyde dehydrogenase 1; AMBRA1: autophagy and beclin 1 regulator 1; AML: acute myeloid leukemia; AMPK: adenosine monophosphate-activated protein kinase; ATG: autophagy-related; Axin1: axis inhibition protein 1; BCR-ABL: breakpoint cluster region-Abelson (leukemia virus); BECN1: beclin1; BNIP3: Bcl-2-interacting protein 3; BNIP3L: Bcl-2-interacting protein 3 ligand; c-Myc: cellular myelocytomatosis, transcription factor, oncogene; CD133: prominin-1; CD200: OX-2 membrane glycoprotein; CD24: heat-stable antigen; CD44: homing cell adhesion molecule; CD90: Thy1; CDK6: cyclin-dependent kinase 6; cGAS: cyclic GMP-AMP synthase; CircRNA: circular RNA; CMA: chaperone-mediated autophagy; CML: chronic myelogenous leukemia; CNSC: cortical neural SC; CPEB1: cytoplasmic polyadenylation element binding protein 1; Cripto-1: cryptic family protein 1; CRC: colorectal cancer; CSC: cancer stem cell; CXCR4: C-X-C chemokine receptor type; DDR: DNA damage repair; DNA: deoxyribonucleic acid; DNMT3L: DNA (cytosine-5)-methyltransferase 3-like; DPSC: dental pulp SC; ECM: extracellular matrix; EMT: epithelial–mesenchymal transition; EP300: E1A-associated protein p300, histone acetyltransferase p300; Eed: embryonic ectoderm development; EPC: endothelial progenitor cell; EpCAM: epithelial-specific marker; ESC: embryonic stem cells; ESCC: esophageal squamous cell carcinoma; EZH2: enhancer of zeste 2; FLT3-ITD: fms-like tyrosine kinase 3-internal tandem duplication mutations; FoxO1: forkhead box protein O1; GABARAP: gamma-aminobutyric acid receptor-associated protein; GAPDH: glyceraldehyde-3-phosphate dehydrogenase; GFAP: glial fibrillary acidic protein; GSC: glioma SC; GSK-3β: glycogen synthase kinase-3β; H3K18ac: histone H3 with an acetylated lysine 18; H3K4me3: trimethylation of lysine 4 on histone H3 protein subunit; H3K56ac: histone H3 with an acetylated lysine 56; H3K9ac: histone H3 with an acetylated lysine 9; H4K16ac: histone H4 with an acetylated lysine 16; HDAC: histone deacetylase; HSC: hematopoietic stem cell; iPSC: induced-pluripotent stem cell; JAK2: Janus kinase 2; Ku70: ATP-dependent DNA helicase II, 70 kDa subunit; LAMP1: lysosomal-associated membrane protein 1; LGR5: leucine-rich repeat-containing G-protein coupled receptor 5; Lin28: lin-28 homolog A; LINE1: long interspersed nuclear element-1; lncRNA: long non-coding RNA; LSC: leukemia SC; MAP1LC3: microtubule-associated protein 1 light chain 3; Mat2a: methionine adenosyltransferase 2A; MiR: microRNA; MiT/TFE: family of transcription factors; mRNA: messenger ribonucleic acid; MSC: mesenchymal SC; mtDNA: mitochondrial DNA; mTORC1: mammalian/mechanistic target of rapamycin complex 1; NAD: nicotinamide adenine dinucleotide; Nanog: homeobox protein, transcription factor; NBR1: neighbor of Brca1 gene; NFATc1: nuclear factor of activated T cells 1; NFkB: nuclear factor kappa light chain enhancer of activated B cells; NICD: Notch intracellular domain; NLRP3: NLR family pyrin domain containing 3; NRF2: nuclear factor erythroid 2–related factor 2; NSCLC: non-small-cell lung cancer; OCT4: octamer-binding transcription factor 4; OSX: osterix, transcription factor Sp7; OXPHOS: oxidative phosphorylation; PA28c: proteasome activator 28; PE: phosphatidylethanolamine; PIK3C3 (VPS34): Phosphatidylinositol 3-kinase catalytic subunit type 3; PINK1: PTEN-induced kinase 1; PLXDC2-OT: plexin domain containing 2 lncRNA; PNPLA2: Patatin-like phospholipase domain containing 2; PP2A: protein phosphatase 2A; PPARα: peroxisome proliferator-activated receptor α; PPARGC1A (PGC-1A): peroxisome proliferator-activated receptor gamma coactivator 1-alpha; PRC4: polycomb repressive complex 4; PRKA: protein kinase, AMP-activated; PtdIns3P: phosphatidylinositol 3-phosphate; RAR: retinoic acid receptor; RB1CC1/FIP200: RB1 inducible coiled-coil 1; RBM6: RNA-binding motif protein 6; RNA: ribonucleic acid; ROS: reactive oxygen species; rRNA: ribosomal RNA; SAM: S-Adenosyl methionine; SC: stem cell; Sir2: Silent Information Regulator 2; SIRT: sirtuin; Smpdl3b: sphingomyelinase phosphodiesterase like 3b; SOD2: superoxide dismutase 2; Sox2: SRY (sex-determining region Y)-box 2; SQSTM1: sequestosome-1; Src: SRC proto-oncogene, non-receptor tyrosine kinase; SSC: spermatogonial SC; STING: stimulator of interferon genes; Suz12: suppressor of Zeste 12; TAG: triacylglycerols; Tert: telomerase reverse transcriptase; Tet1/2: ten-eleven translocation 1/2; TFEB: transcription factor EB; TKI: tyrosine kinase inhibitor; tRNA: transfer RNA; Ub: ubiquitin; ULK1: Unc-51-like kinase 1; Usp22: ubiquitin-specific peptidase 22; UV: ultraviolet; WIPI2: WD repeat domain, phosphoinositide interacting 2; Wnt: wingless-related integration site; β-TrCP: β-transducin repeat-containing protein.
